# Nature of the Interaction of Pyridines with OCS. A Theoretical Investigation

**DOI:** 10.3390/molecules25020416

**Published:** 2020-01-19

**Authors:** Sumitra Bhattarai, Dipankar Sutradhar, Asit K. Chandra, Therese Zeegers-Huyskens

**Affiliations:** 1 Department of Chemistry, North-Eastern Hill University, Shillong 793022, India; sumitrabhattarai@gmail.com (S.B.); dipankarsutradhar09@gmail.com (D.S.); 2 Department of Chemistry, University of Leuven, Leuven 3000, Belgium

**Keywords:** substituted pyridines, OCS, chalcogen bonds, tetrel bonds, π-bonds, vibrational shifts

## Abstract

Ab initio calculations were carried out to investigate the interaction between *para*-substituted pyridines (X-C_5_H_4_N, X=NH_2_, CH_3_, H, CN, NO_2_) and OCS. Three stable structures of pyridine.OCS complexes were detected at the MP2=full/aug-cc-pVDZ level. The **A** structure is characterized by N…S chalcogen bonds and has binding energies between −9.58 and −12.24 kJ/mol. The **B** structure is bonded by N…C tetrel bond and has binding energies between −10.78 and −11.81 kJ/mol. The **C** structure is characterized by π-interaction and has binding energies between −10.76 and −13.33 kJ/mol. The properties of the systems were analyzed by AIM, NBO, and SAPT calculations. The role of the electrostatic potential of the pyridines on the properties of the systems is outlined. The frequency shift of relevant vibrational modes is analyzed.

## 1. Introduction

Hydrogen bonds, without doubt, are the most studied intermolecular interactions today [1,2]. Other molecular complexes although discovered 150 years ago, have received considerably less attention. However, in the last few decades, there has been an increased attention in “charge transfer” complexes, following the nomenclature of Mulliken [3,4]. These complexes involve the interaction of the XB…YZ type where Y possesses one or more electron pairs. They have been classified into halogen bonds where B is a halogen [5,6,7,8,9,10,11,12,13,14,15], chalcogen bonds where B = O, S, Se [16,17,18,19,20,21,22,23,24,25,26,27,28,29,30,31,32,33,34], and more recently, tetrel bonds where B = C, Si [35,36,37,38,39,40,41,42]. These bonds are unusual in that they involve a close approach of two electronegative atoms such as Cl, O, C on the one hand, and Y atoms such as N on the other hand. This peculiar bond formation was discussed by Politzer et al. [43,44,45] who showed that although halogen atoms carry partial negative charges, they have a region of positive electrostatic potential at the head of the halogen atom in the opposite direction of the XB axis. This region was called a σ-hole.

In a recent work [46], where the interaction between *para*-substituted pyridine derivatives and CS_2_ was investigated, the versatility of the CS_2_ molecule which forms with pyridines four types of complexes with different structure was demonstrated. This present work deals with the interaction of *para*-substituted pyridines with OCS. It must be noted that the OCS interaction with the Cl^−^ anion [32] and with NH_3_, H_2_O, H_2_S has been investigated. The interaction of OCS with N derivatives [34,47] has also been discussed recently. The interaction of guest molecules with pyridines is interesting to investigate because the substitution allows one to modulate their basic strength, as for example in their interaction with atomic chlorine [48].

To the best of our knowledge, no experimental data on pyridines-OCS interaction is available in the literature and the present study is purely theoretical. The purpose of this work is to study the interaction between *para*-substituted pyridines and OCS and to compare it with pyridines.CS_2_ systems for gaining knowledge about the modulation of the strength of chalcogen and tetrel bonds with the change in the strength of the electrostatic potential of the electron donor and acceptor atoms. Let us also observe that the S derivatives are one of the main pollutants in the atmosphere [49,50] and a large quantity of CS_2_ and OCS are released into the atmosphere from the ocean. From this point of view, knowledge of its interaction with other molecules may be helpful in understanding its atmospheric chemistry. Our study involves the optimization of the geometry of the different structures. Detailed natural bond orbital (NBO) and atoms-in-molecules (AIM) analysis along with symmetry adapted perturbation theory (SAPT) results are presented. The correlation between the properties of the complexes and the electrostatic potential of the components can be very useful in discussing the nature of the interaction and will be presented in a first step. 

Theoretical calculations were carried out at the MP2/aug-cc-pVTZ//MP2/aug-cc-pVDZ level. Natural bond orbital (NBO) analysis was performed at the wB97XD/aug-cc-pVDZ level on the MP2 optimized geometries. 

## 2. Results and Discussion

### 2.1. Electrostatic Potential of the Components

The values of the molecular electrostatic potentials of the substituted pyridines and OCS which are relevant for the discussion of the results are listed in Table 1. As outlined in a recent work [46], the negative electrostatic potential at the N-atom of substituted pyridines is very sensitive to the substituents, decreasing in the order NH_2_ > CH_3_ > H > F > CN > NO_2_ which is also the order of the p.Hammett constants (−0.660, −0.170, 0.062, 0.660, 0.778) [51].

**Figure 1:**
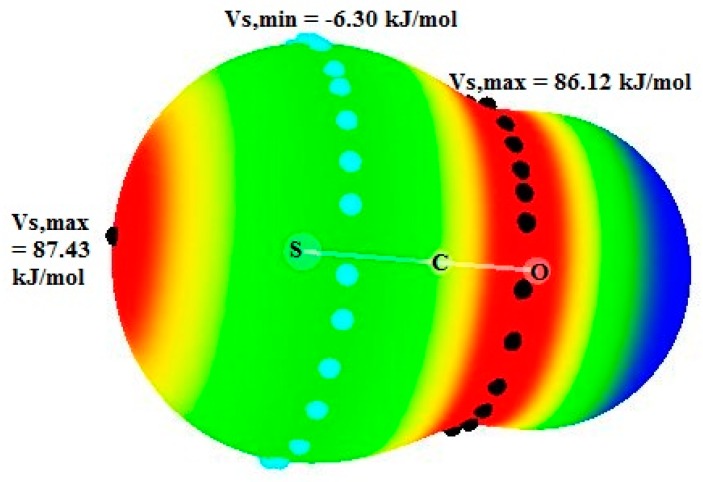
Electrostatic potential diagram of OCS molecule.

Figure 1 illustrates the electrostatic potential of OCS molecule. The S atom of OCS has a σ-hole along the extension of the C=S bond, which is characterized by a V_s,max_ value 87.4 kJ/mol. A positive electrostatic potential belt around the C atom is predicted with a V_s,max_ value of 86.1 kJ/mol. The V_s,max_ values of the S and C atom of OCS are much greater than that for the S-atom (70.9 kJ/mol) and of the C atom (23.1 kJ/mol) of CS_2_ molecule [46] because of the presence of O atom in OCS. Interestingly, the S atom develops a belt of negative electrostatic potential with a V_s,min_ value of −6.3 kJ/mol. From this data, it can be anticipated that the OCS molecule will be able to act as an electron acceptor and as a weak electron donor.

### 2.2. Binding Energies

**Figure 2:**
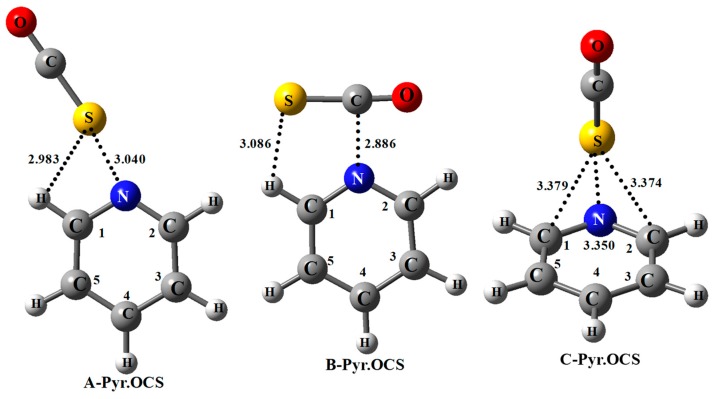
Structures of **A**, **B**, and **C** complexes between *para*-substituted pyridine with OCS. Bond lengths (in Å) correspond to nonsubstituted pyridine complex.

Three stable structures (**A**, **B**, and **C**) are predicted for the pyridine.OCS complexes based on their interaction through chalcogen and tetrel bonds. A fourth structure of the complex is possible where the OCS molecule lies parallel to the pyridine ring and a significant interaction with the substituent is found (Figure S1 in Supplementary Information). This complex is not discussed here as our primary aim is to discuss the nature and modulation of chalcogen and tetrel bonds in these systems with basis properties of the pyridines. The hydrogen bonding interaction between the ortho C–H of pyridine and O atom of OCS was also considered, but binding energy of such complexes were found to be less than 2 kJ/mol. The structures of **A**, **B**, and **C** complexes of pyridine.OCS are illustrated in Figure 2 where the intermolecular distances are indicated for the unsubstituted pyridine.OCS complex. These structures are the most stable ones and are very similar to those recently predicted for the pyridines.CS_2_ complexes [46]. Table 2 reports the binding energies calculated at the MP2=full/aug-cc-pVTZ//aug-cc-pVDZ level.

As illustrated in Figure 2, the structures of the **A**, **B**, and **C** complexes are very different and therefore their characteristics will be discussed in different sections. Let us notice that these structures are very similar to those reported for the same pyridine derivatives complexed with CS_2_ [46].

As mentioned before, there are other stable structures characterized by binding energies between −12.95 and −16.15 kJ/mol. These structures primarily involve the substituents of pyridine and do not reflect the basic properties of the pyridines. As a consequence, they will not be considered in the present work.

### 2.3. A-Complexes of Pyridines.OCS

The **A** complexes are planar or nearly so. The N…S distances are shorter than the sum of the van der Waals radii of N and S (3.35 Å) and are in the range between 3.021 and 3.068 Å. The N…SC chalcogen bond is not linear, and the N…SC angle varies between 167° and 169°, suggesting an interaction between the C1H bond and the S atom. This is in agreement with the intermolecular H…S distances which range between 2.989 Å and 2.964 Å, thus shorter than the sum of the van der Waals distances of H and S (3.0 Å). Let us observe that these distances are decreasing on going from NH_2_-pyridine to NO_2_-pyridine.

The binding energies are rather low, between −12.24 and −9.58 kJ/mol. The **A**-complex of pyr.OCS systems is slightly more stable than the corresponding pyr.CS_2_ complex as expected from the higher V_s max_ value of the S atom in OCS. Similarly, for complexes between pyridines and CS_2_ [46], these energies depend on the proton affinity and the ionization potential of the pyridines (correlation equations are given in **S.I.1** of Supplementary Information). The binding energies are also related to the V_s,min_ of the pyridines (both in kJ/mol):

−ΔE = −0.036 V_s,min_ + 5.55  (r^2^ = 0.964) (1)

For the pyridines–CS_2_ interaction, a slope of −0.029 was calculated. The results of the present work show that the **A**-complexes of pyr.OCS systems are slightly stronger than the corresponding pyr.CS_2_ complex [46], as expected from the higher V_s,max_ value of the S atom in OCS. This is in agreement with results on the interaction with NH_3_ and H_2_O which is somewhat stronger for the OCS than for the CS_2_ interaction; for the weak complexes with PH_3_ and H_2_S, they are of the same order of magnitude [33]. However, for the strong interaction with the Cl^−^ anion, the CS_2_ complexes seem to be stronger than the OCS ones [32]. Contrary to the expectation from the much greater V_s,max_ value of the S atom in OCS compared to CS_2_, the difference of binding energies between pyr.OCS and pyr.CS_2_ complexes is very small. The CH…S H–bonding interaction is weaker for the pyr.OCS complex because of the presence of electronegative O-atom in OCS. In fact, AIM results do not reveal BCPs for CH…S H–bonding in pyr.OCS complexes except for the NO_2_-pyr.OCS complex.

Let us compare the binding energies of the **A**-pyridines.OCS and **A**-pyridines.CS_2_ complexes with the electrostatic potential of the interacting atoms. The binding energies of the NH_2_-pyr.OCS and NO_2_-pyr.OCS complexes are equal to −12.24 and −9.58 kJ/mol and the difference between the V_s,min_ values is about 70 kJ/mol (Table 1). The NO_2_-pyr.OCS and NO_2_-pyr.CS_2_ systems have binding energies of −9.58 and −9.34 kJ/mol, the difference between the electrostatic potential of the S atom being about 16 kJ/mol. From these data, it can be concluded that, in the present cases, the electrostatic potential of the electron donor is more important than the electrostatic potential of the electron acceptor in determining the binding energies. Results on other interactions with PH_3_, H_2_S, [33] and Cl^−^ [32] are in agreement with this conclusion.

**Figure 3:**
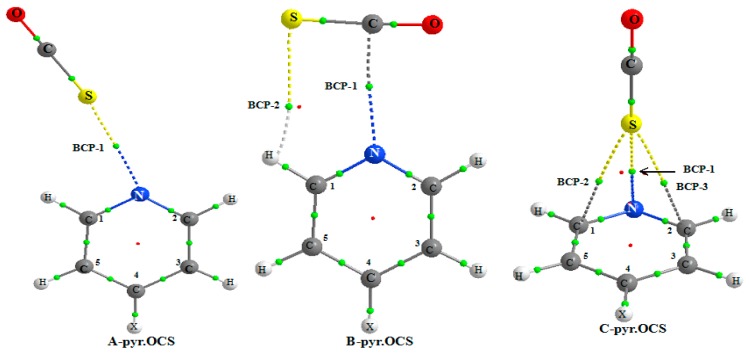
Atoms in Molecules (AIM) picture for the **A**, **B**, and **C** complexes of pyridine.OCS showing the existence of bond critical point (BCP).

Table 3 reports the different parameters obtained from an AIM analysis. Let us observe that the AIM results have been questioned for weak interactions [52]. The results of the present work show that the AIM parameters are well correlated with other parameters describing the nature of the interaction (see further discussion). Figure 3 shows the AIM picture for the complex of non-substituted pyridine and the existence of BCPs for the three **A**, **B**, and **C** systems.

For all the **A** complexes, a BCP-1 is obtained between the N and S atoms. A second BCP-2 is predicted between the S and H1 atom, only for the NO_2_-pyr.OCS complex. This indicates that the S…H1 interaction is stronger in this system, as expected from the higher acidity of the ortho C–H bond due to the presence of a strong electron withdrawing group at pyridine. The S…HC1 hydrogen bond is far from linear, the S…HC1 angle increasing slightly from 106.4° to 108.5° from the NH_2_-pyr.OCS to the NO_2_-pyr.OCS system. This also suggests that the S…HC1 hydrogen bond is slightly stronger in this last system.

The values of electron density and its Laplacian fulfill the criteria expected for closed–shell interaction [53,54]. The ρ(r_c_) values for BCP-1 are comprised between 0.0130 and 0.0117 a.u., whereas BCP-2 could be detected only for the NO_2_-pyr.OCS interaction. The ρ(r_c_) values for BCP-1 in pyridine.OCS complexes are somewhat greater than those observed for the pyridine.CS_2_ complexes, thus indicating that the N…S bond is stronger in the former system. The positive values of H(r_c_) and G(r_c_)/ρ(r_c_) ratio further indicate further that the N…S interaction is primarily electrostatic in nature. The binding energy (in kJ/mol) of the pyridine.OCS complexes is strongly correlated with the ρ(r_c_) values (in me) for BCP-1 as given below.

−ΔE = −14.23 + 2.03 ρ(r_c_)  (r^2^ = 0.995) (2)

The NBO analysis provides interesting data on the nature of the interaction in the **A** complexes. Table 4 reports the NBO charges on the S, C, and O atoms of OCS, the charge transfer from pyridines to OCS along with the variation of the C=O and C=S distances.

Let us observe that the charge transfer (CT) in pyr.OCS is significantly larger than the CT in pyridines.CS_2_ systems where the values are comprised between 7.4 and 3.4 me. This is obviously due to the presence of stronger σ-hole at the S atom of OCS compared to CS_2_.

The charge transfer (me) is related to the V_s,min_ values of the electrostatic potential (a.u) of pyridines by the equation:

CT = −0.037 V_s,min_ + 3.33  (r^2^ = 0.933) (3)

Table 5 reports the most relevant hyperconjugation energies in the **A** systems.

The NBO analysis confirms the existence of both N…S and C1H…S interactions, the N…S interaction being the stronger one. This is in agreement with the correlation between the binding energies and the hyperconjugation energies from the LP(N) to σ*(C=S):

−ΔE = 1.16 [LP(N)→σ*(C=S)] + 0.786  (r^2^ = 0.993) (4)

Comparison of the data of Table 4 and Table 5 shows that in agreement with the LP(N)→σ*(C=S) charge transfer, the charges on the S atom are decreasing. The calculations also reveal a charge transfer from the LP(S) to σ*(C1H); this charge transfer increases from NH_2_-pyridine to NO_2_-pyridine. This increase is in agreement with the decrease of the intermolecular S…HC1 distance and is most probably due to the increase in acidity of the C1H bond.

It is surprising that despite the fact that the LP(N)→σ*(C=S) charge transfer is sensitive to the substitution, the elongation of the C=S bond remains more or less constant. A possible explanation is that the increase of σ*(C=S) occupation is nearly compensated by a decrease in π*(C=S) occupation [23]. Another explanation is an electrostatic attractive interaction between the C and S atoms [55].

A SAPT analysis was performed to gain further insight into the nature of the interaction. The results are reported in **S.I.2** of Supplementary Information. The interaction energies are slightly higher than the binding energies calculated at the MP2 level because of distortion energy and higher order electron correlation at the SAPT level. This analysis shows that the electrostatic interaction decreases from NH_2_-pyridine (−21.11 kJ/mol) to NO_2_-pyridine (−16.58 kJ/mol) as expected from the V_s,min_ values. However, the contribution of the electrostatic energy to the total energy decreases slightly from NH_2_-pyridine (46%) to NO_2_-pyridine (44%) while the contribution of dispersion energy increases, from 39% to 44%.

### 2.4. B-Complexes of Pyridines.OCS

As shown in Figure 2, the **B** complex is formed due to the interaction of the positive electrostatic potential of the C atom of OCS with the N atom of pyridine, resulting in a weak N…C tetrel bond. The binding energies of pyridine.OCS **B** complexes range between −10.78 and −11.81 kJ/mol and are related to the PA and IP of the pyridines (correlation equations are given in **S.I.3** of Supplementary Information). In the **B** structure (Figure 2), the N…C distances are shorter than the sum of the van der Waals radii (3.25 Å) and range between 2.869 and 2.907 Å. These distances indicate the formation of N…C tetrel bonds. The binding energies decrease with increasing N…C intermolecular distances. The binding energies of the **B** complexes of pyr.OCS are almost 2 kJ/mol greater than that for the corresponding pyr.CS_2_ complexes, because of stronger π-hole at the C-atom of OCS compared to CS_2_. In fact, for pyridine, F-pyridine, NC-pyridine, and NO_2_-pyridine, the **B** complex is slightly more stable than the corresponding **A** complex; which is in complete contrast with the **B** complexes of pyridines.CS_2_ where the **B** complex was found to be the weakest [46]. Although the S…HC1 distances are relatively long (between 3.066 and 3.127 Å), our further analysis suggests the formation of S…HC1 hydrogen bonds. This is in agreement with the small elongation of the C1H bond comprised between 0.33 and 0.47 Å. 

The basic structure of the **B** complex of pyridines.CS_2_ and pyridines.OCS systems is the same. Both involve the formation of N…C tetrel bonds. The fundamental difference is the direction of the charge transfer which in the pyridines.CS_2_ systems is directed from CS_2_ to pyridines and in the pyridines.OCS systems from pyridines to OCS. This may be due to the fact that the electrostatic potential around the C atom is equal to 86.12 kJ/mol in OCS, which is much higher than this potential in CS_2_ (23.10 kJ/mol) [46]. This agrees with the charges on the C atom, respectively equal to 0.468 e in OCS and −0.342 e in isolated CS_2_.

Table 6 reports the results obtained from the AIM analysis. Two BCPs (as illustrated in Figure 3) are predicted, the first one between the N and S atoms (BCP-1) and the second one (BCP-2) between the S and H1 atoms (at the exception of CH_3_-pyridine). The values of the electron density and its Laplacian are larger for BCP-1 than for BCP-2. Furthermore, the binding energy is found to be linearly correlated with the ρ(r_c_) value of BCP-1. These two factors indicate that the N...C tetrel bond is the primary interaction in **B** complex. The ρ(r_c_) values of BCP-1 of pyr.OCS and pyr.CS_2_ complexes range between 13.7 to 12.7 and 10.2 to 9.8 me respectively, indicating that the tetrel bond is stronger for the former complex.

Table 7 reports the charges on the S, C, and O atoms, the charge transfer from pyridine to OCS along with the variations of the C=S and C=O distances for the **B** complex of pyridines.OCS systems.

These results indicate a moderate charge transfer, from 4.2 to 8.5 me. Surprisingly, the positive charges on the C atom are increasing in complex formation, whereas the O and S atoms of OCS are gaining electron. The elongation of the C=S bond and the contraction of the C=O bond remains approximately the same for all the complexes.

Table 8 reports the most important hyperconjugation energies in the **B** complexes. There is in all the systems a non-negligible hyperconjugation to the Rydberg orbitals of the N atom from σ (C–O) (4 kJ/mol) and from σ(C–S) bonds (2.5 kJ/mol). The values of the binding energies are related to the hyperconjugation energies to the σ* (CO) orbitals by the relation:

−ΔE = 0.289 E^2^[(LP(N)→σ*(CO)] + 7.05  (r^2^ = 0.973) (5)

This indicates that the N atom is the main interaction site. Despite the relatively long S…HC1 distances, the NBO analysis reveals a non-negligible charge transfer to the σ*(C1H) orbital in agreement with the elongation of the C1H bond of c.a 0.35 mÅ for all the complexes. This charge transfer is about the same as in the **A** complexes.

No CH…C hydrogen bonds between the CH bond of pyridines and the C atom of OCS could be detected in the present system. This bond was predicted in the complexes between diazines (pyrazine, pyrimidine, and pyridazine) and CS_2_ [28]. These interactions are weak (−ΔE between 3.1 and 5.3 kJ/mol). The formation of CH…C hydrogen bonds in diazines. CS_2_ systems can be explained by the presence of two N atoms in the heterocyclic ring which increases the acidity of the CH bond by delocalization of the two N LPs of diazines to the σ*(CH) orbital.

We also want to mention the work recently published [34] on the interaction between substituted azine molecules HN(CH)SX (X = F, NC, Cl, CN, CCH, H) and OCS. Calculations performed at the MP2 level with the aug-cc-pVTZ basis set indicate that the complex is cyclic. A tetrel bond N…C is formed between the N of the azine molecule and the C atom of OCS and a chalcogen bond O…S is formed between the O atom of OCS and the S atom of the azine molecule. In contrast with the present results, the S atom of OCS is not involved in the interaction. A charge transfer is calculated from the LP of the N atom to the π*(C=S) and π*(C=O) orbitals.

The results of SAPT calculations are reported in **S.I.4** of the Supplementary Information. They are very similar to the results obtained for the **A** complexes. The contribution of the electrostatic energy remains almost constant (44%–45%) of the total energy and the contribution of the dispersion energy slightly increases from NH_2_-pyridine (43%) to NO_2_-pyridine (46%).

### 2.5. C-Complexes of Pyridines.OCS

In this case of pyridine.OCS interaction, the OCS molecule is nearly perpendicular to the pyridine ring, the NSCO dihedral angle being equal to 179°. The binding energies are comprised between −13.33 kJ/mol and −10.76 kJ/mol.

For the complex between pyridine and OCS, the AIM analysis indicates the existence of a BCP between the N and S atoms and between the S and C1 and C2 atom. For other systems, the AIM picture indicates the existence of a BCP between the C4 or C5 atoms and the S atom. The electron density fluctuates between 0.0068 and 0.0081 a.u. and its Laplacian between 0.0232 and 0.0282 a.u. This π interaction induces a weak charge transfer from pyridine to OCS, ranging from 6.84 to 4.35 me.

The correlations between binding energies and PA or IP of the pyridines are characterized by worse correlation coefficients (given in **S.I.5** of Supplementary Information). This is also the case for the correlation between the binding energies and V_s,min_ values:

−ΔE = −0.037 V_s,min_ + 6.47  (r^2^ = 0.718) (6)

This suggests that the contribution of the N…S interaction to the total binding energy is smaller in these complexes.

NBO results of the **C** complexes are listed in Table 9. The CT for these complexes is quite moderate and range between 4.4 and 6.8 me. On complex formation, the electron density on the C1 and C2 atoms of pyridine ring increases slightly whereas the charge on N atom of pyridine almost remains unchanged. The positive charge on the S atom of the OCS molecule increases for the –NH_2_, −CH_3_, −H, and –F substituted complexes whereas it decreases for the –CN and –NO_2_ substituted complexes. The negative charge on the O atom of the OCS molecule increases to some extent (between 0.02 and 15 me) on complex formation with the exception of –NO_2_ complex where a decrease in electron density of the O atom has been observed. Moreover, complex formation results in a very negligible increase in the C=S bond length except for the –NH_2_ (0.33 mÅ) and –H (0.08 mÅ) substituted complexes where a slight contraction of the C=S bond has been predicted. The C=O bond distances also gets elongated in all the complexes within a margin ranging between 0.09 and 2.37 mÅ. As mentioned in Table 9, the major source of stabilization for these complexes comes from the π(C2-C3)→σ*(C-S), π(C4-C5)→σ*(C-S), and π(C1-N)→σ*(C-S) hyperconjugation energies but the small contribution from π(C2-C3)→π*(C-O) orbital interaction cannot be overlooked. It should be noted that the occupation of the σ*(C-S) orbital in isolated OCS is 0.0102 e. This occupation increases slightly in all the complexes as mentioned in Table 9, resulting in a small increase in the C=S bond length.

The results of a SAPT analysis are reported in **S.I.6** of the Supplementary Information. In the case of **C** complexes, we observe a great difference in the nature of interaction with **A** and **B** complexes. The contribution of the electrostatic energy to the total binding energy fluctuates between 30% (NH_2_-pyr) and 25% (NO_2_-pyr); whereas the contribution of the dispersion energy ranges between 58% and 65% and is much larger than that for the **A** and **B** complexes. This was to be expected for an interaction involving mainly π-electrons.

### 2.6. Vibrational Data

Some relevant vibrational data will be discussed here. Table 10 reports the frequency shifts of the ν(C=O) and ν(C=S) vibrations of the **A**, **B**, and **C** complexes. 

For the much stronger OCS…Cl^−^ interaction, Δν(C=S) and Δν (C=O) values of 31.4 and 63 cm^−1^ were reported [32].

From these results and from comparing Table 4 and Table 7, it appears that the red shift in ν(C=S) in **A** and **B** complexes of pyridine.OCS is due to the elongation of the C=S bond owing to the increase in the σ*(C-S) population. The Δν(C=S) is negligible for the **C** complexes. For the **A** and **C** complexes, the Δν(C=O) are negative and corresponds to an elongation of the C=O bond. On the other hand, the positive Δν(C=O) values for the **B** complexes correspond to a contraction of the C=O bond upon complex formation.

The deformation vibration δ(OCS) is degenerate in the isolated OCS molecule and its frequency is predicted at 513.3 cm^−1^. In complexes **A** and **C**, this vibration remains degenerate and is blueshifted between 17.7 and 14.5 cm^−1^ in **A** complexes and between 8.5 and 4.1 cm^−1^ in **C** complexes. In the **B** complexes, the degeneracy is removed and the vibration is split into two components. The first component is redshifted between 32.3 cm^−1^ and 24.3 cm^−1^; the second component is blueshifted by small amounts, between 3.7 and 2.7 cm^−1^.

The pyridine vibrations are shifted by small amounts, between 1 and 5 cm^−1^. Interestingly, the vibration predicted at 3197 cm^−1^ which is predominantly the v(CH) vibration is calculated at 3202 cm^−1^ in the **A**-pyr.OCS complex. The red shift of 5 cm^−1^ can be explained by the formation of a weak S…H1C hydrogen bond and the charge transfer to the σ*(C1H) orbital which elongates the C1H bond. In contrast, in the pyridine.Cl^−^ complex, the ν(C1H) vibration is blueshifted by 25 cm^−^1 [32]; this blue shift can be explained by a decrease of the anomeric effect because the LP of the N atom is partially involved in the formation of the intermolecular N…Cl^−^ bond.

## 3. Computational Methodology

The geometries of the isolated *para*-substituted pyridines and their complexes with OCS molecule were optimized at the MP2=full/aug-cc-pVDZ level of theory. Harmonic frequency calculations were carried at the same level to characterize the stationary points. The binding energies are refined further from the single point energy calculations at the MP2/aug-cc-pVTZ//MP2/aug-cc-pVDZ level. This method has been applied in several recent studies of noncovalent interaction and found to be quite reliable [46,56,57,58]. The binding energy of the complexes was calculated using the usual super molecular approach where the energies of the optimized monomers are subtracted from the energy of the complex. The interaction energy of the complexes includes zero-point energy (ZPE) and the basis set superposition error (BSSE) correction computed by the counterpoise method [59]. Natural bond orbital (NBO) [60] analysis was performed at the wB97XD/aug-cc-pVDZ level on the MP2 optimized geometries using Gaussian NBO 3.1, to obtain ideas about the charges on the individual atoms, orbital occupancies, hyperconjugation energies, etc. Atoms in molecules (AIM) [61] analysis was carried out at the MP2/aug-cc-pVDZ level using the AIMALL program [62] to gain better understanding of the nature of interaction. The electrostatic potentials on the isolated substituted pyridines and OCS molecule were calculated at the MP2/aug-cc-pVDZ level using the wave function analysis and surface analysis suite (WFA-SAS) [63]. The energy decomposition analysis was carried out employing symmetry adapted perturbation theory (SAPT) using Psi4 program [64,65]. The SAPT calculations were performed at the SAPT2+/aug-cc-pVTZ level using the MP2 optimized geometries. All electronic structure calculations were carried out using G09 [66] suite of program.

## 4. Conclusions

This work presents the results of theoretical calculation on the interaction of para-substituted-pyridines with OCS. The calculations are carried out at the MP2=full/aug-cc-pVTZ//aug-cc-pVDZ level. The nature of the interaction is discussed by using the results of AIM, NBO, and SAPT calculations. The main conclusions are outlined below:

(1) In the first part, the electrostatic potential of the pyridines and OCS are reported. This potential plays an important role in the interpretation of the results.

(2) The optimization of the structures indicates three stable **A**, **B**, and **C** complexes comprising of either chalcogen or tetrel bonds. These structures are similar to the structures reported for the pyridines.CS_2_ systems.

(3) The **A** complexes are bonded by N…S chalcogen bonds. The binding energies ranging between −8.95 and −12.24 kJ/mol are correlated to the electrostatic potential of the pyridines. The AIM and NBO analysis reveal the existence of weaker S…H interactions. The charge transfer from pyridine to OCS is moderate and increase with the electrostatic potential of the pyridines. The binding energies of the **A** complexes of pyridines.OCS systems are only 2%–5% larger than the corresponding pyridines.CS_2_ complexes.

(4) The **B** complexes are bonded by N…C tetrel bonds. The binding energies range from −10.78 to −11.81 kJ/mol. Weak CH…S interactions also stabilize the structure. In contrast with the pyridines.CS_2_ complexes, the charge transfer occurs from pyridines to OCS and not from OCS to pyridines. The binding energies of the **B** complexes of pyridines.OCS systems are 20%–26% larger than the corresponding pyridines.CS_2_ complexes [46] owing to the stronger π-hole at the C atom of OCS. The N…C tetrel bond is found to be almost as strong as the N…S chalcogen bond in pyr.OCS complexes.

(5) The **C** complexes are characterized by N…S, C1…S, and C2…S interactions which varies with the inclusion of substituents. The binding energies of these complexes vary between −10.76 and −13.33 kJ/mol. For these complexes, the involvement of electrons of pyridine in the complex formation can be demonstrated from the π(C2-C3)→σ*(C-S), π(C4-C5)→σ*(C-S), and π(C1-N)→σ*(C-S) orbital interactions. Contrary to the **A** and **B** complexes, the **C** complexes of pyridines.OCS systems are found to be weaker than the similar complexes for pyridines.CS_2_ systems.

(6) SAPT analysis shows that the electrostatic interaction is the dominant force for the **A** and **B** complexes of pyridines.OCS; whereas dispersion energy plays the most vital role for the **C** complex.

(7) A comparison between the A-CS_2_.pyridines and A-OCS.pyridines complexes suggest that the electrostatic potential of the electron donors (pyridines) has a greater influence on the interaction energies than the electrostatic potential of the electron acceptors (CS_2_ and OCS).

## Figures and Tables

**Table 1:** Electrostatic potentials (V in kJ/mol) of pyridines ^a^ and OCS calculated at the MP2=full/aug-cc-PVDZ level.

Pyridines	V_s,min_	OCS	V
NH_2_-pyridine	−178.3	V_s,max_ (S)	87.4
CH_3_-pyridine	−168.3	V_s,min_ (S)	−6.3
pyridine	−159.9	V_s,max_ ( C)	86.1
F-pyridine	−143.6	V_s, min_ (O)	−60.9
CN-pyridine	−115.8		
NO_2_-pyridine	−108.4		

^a^ Fromreference [46]. OCS = carbonyl sulfide.

**Table 2:** Binding energies (kJ/mol) of the **A**, **B**, and **C** complexes between substituted pyridines and OCS.

Systems	A-Complex	B-Complex	C-Complex
NH_2_-pyr.OCS	−12.24	−11.81	−13.33
CH_3_-pyr.OCS	−11.53	−11.53	−13.39
Pyr.OCS	−11.03	−11.23	−11.56
F-pyr.OCS	−10.56	−11.04	−10.88
CN-pyr.OCS	−9.69	−10.79	−10.94
NO_2_-pyr.OCS	−9.58	−10.77	−10.76

**Table 3:** Electron density ρ(r_c_), Laplacian of electron density ∇^2^(ρ), total energy H(r_c_) and G(r_c_)/ρ(r_c_), values for bond critical point (BCP-1) and BCP-2 for the **A**-pyr.OCS complexes. All values are in a.u.

Systems	ρ(r_c_)	∇^2^(ρ)	H(r_c_)	G(r_c_)/ρ(r_c_)
NH_2_-pyr.OCS				
BCP-1	0.0130	0.0401	0.0011	0.6846
CH_3_-pyr.OCS				
BCP-1	0.0127	0.0392	0.0011	0.6850
Pyr.OCS				
BCP-1	0.125	0.0387	0.0011	0.6880
F-pyr.OCS				
BCP-1	0.0122	0.0380	0.0011	0.6886
CN-pyr.OCS				
BCP-1	0.0118	0.0368	0.0011	0.6864
NO_2_-pyr.OCS				
BCP-1	0.0117	0.0366	0.0011	0.6923
BCP-2	0.0065	0.0254	0.0012	0.7846

**Table 4:** Natural Bond Orbital (NBO) charges (e) on the S, C, and O atoms of OCS, charge transfer (me) from pyridines to OCS, and variation of the C=O and C=S distances (mÅ) in the **A** complexes ^a,b^.

System	q(S)	q(C)	q(O)	CT	Δr(C=O)	Δr(C=S)
NH_2_-pyr.OCS	0.0258	0.4554	−0.4917	10.4	+2.57	+3.78
CH_3_-pyr.OCS	0.0230	0.4572	−0.4897	9.4	+2.28	+3.75
Pyr.OCS	0.0207	0.4585	−0.4883	9.0	+2.11	+3.75
F-pyr.OCS	0.0165	0.4608	−0.4855	8.4	+1.76	+3.77
CN-pyr.OCS	0.0093	0.4649	−0.4908	7.7	+1.18	+3.86
NO_2_-pyr.OCS	0.0082	0.4655	−0.4800	7.5	+1.15	+3.80

^a^ In isolated OCS, q(S) = 0.0034e, q(C) = 0.4686 e, q(O) = −0.4720 e. ^b^ In isolated OCS, r(C=O) = 1.179 Å, r(C=S) = 1.581 Å.

**Table 5:** Most predominant hyperconjugation energies (kJ/mol) in the **A** complexes of pyridines.OCS.

Systems	E^2^ [LP(N)→σ*(C=S)]	E^2^ [LP(S)→σ*(C1H)]
NH_2_-pyr.OCS	9.75	2.97
CH_3_-pyr.OCS	9.25	3.18
Pyr.OCS	8.91	3.22
F-pyr.OCS	8.45	3.35
CN-pyr.OCS	7.66	3.64
NO_2_-pyr.OCS	7.49	3.77

**Table 6:** Electron density values ρ(r_c_), Laplacian of electron density ∇^2^(ρ), total local energy H(r_c_) and G(r_c_)/ρ(r_c_) values for the **B** complexes. All data are in a.u.

Systems	ρ(r_c_)	∇^2^(ρ)	H(r_c_)	G(r_c_)/ρ(r_c_)
NH_2_-pyr.OCS				
BCP-1	0.0137	0.0418	0.0008	0.7007
BCP-2	0.0058	0.0177	0.0008	0.6034
CH_3_-pyr.OCS				
BCP-1	0.0135	0.0416	0.0008	0.7037
Pyr.OCS				
BCP-1	0.0133	0.0407	0.0009	0.6992
BCP-2	0.0055	0.0170	0.0008	0.6182
F-pyr.OCS				
BCP-1	0.0130	0.0399	0.0009	0.7000
BCP-2	0.0057	0.0172	0.0008	0.5965
CN-pyr.OCS				
BCP-1	0.0128	0.0396	0.0009	0.7031
BCP-2	0.0054	0.0166	0.0008	0.6111
NO_2_-pyr.OCS				
BCP-1	0.0127	0.0392	0.0009	0.7008
BCP-2	0.0056	0.0171	0.0008	0.6071

**Table 7:** NBO charges on the S, C, and O atoms (e), charge transfer from pyridine to OCS (me) and variation of the C=S and C=O distances (mÅ) in the **B**-pyridines.OCS complexes.

Systems	q(S)	q(C)	q(O)	CT	Δr(C=S)	Δr(C=O)
NH_2_-pyr.OCS	−0.0301	0.5012	−0.4796	8.5	2.45	−1.74
CH_3_-pyr.OCS	−0.0269	0.4994	−0.4802	7.7	2.13	−1.55
Pyr.OCS	−0.0266	0.4987	−0.4792	7.1	2.19	−1.58
F-pyr.OCS	−0.0259	0.4983	−0.4784	6.0	2.17	−1.65
CN-pyr.OCS	−0.0222	0.4959	−0.4784	4.6	2.04	−1.49
NO_2_-pyr.OCS	−0.0221	0.4956	−0.4777	4.2	2.15	−1.56

**Table 8:** Most important hyperconjugation energies (kJ/mol) in the **B** complexes between pyridines and OCS.

Systems	E^2^ [LP(N)→σ*(CO)]	E^2^ [LP(S)→σ*(C1H)]
NH_2_-pyr.OCS	16.32	3.47
CH_3_-pyr.OCS	15.56	2.89
Pyr.OCS	14.85	3.35
F-pyr.OCS	13.93	3.60
CN-pyr.OCS	13.05	3.39
NO_2_-pyr.OCS	12.72	3.68

**Table 9:** NBO results of **C** complexes between *para*-substituted pyridine and OCS.

Parameters	–NH_2_	–CH_3_	–H	–F	–CN	–NO_2_
CT (me)	6.8	5.6	6.4	5.8	4.5	4.4
σ*(C-S) (me)	13.3	12.7	13.6	13.7	13.4	12.1
Δq(C1) (me)	−3.93	−0.83	−5.93	−5.76	−4.30	−4.02
Δq(C2) (me)	−3.98	−3.94	−5.46	−5.42	−4.03	−3.79
Δq(S) (me)	+15.34	+6.24	+9.6	+1.08	−11.94	−15.09
Δr(C-S) (mÅ)	+2.37	+1.55	+1.76	+1.15	+0.28	+0.09
Δr(C-O) (mÅ)	−0.33	+0.82	−0.08	+0.19	+0.61	+0.71
π(C2-C3)→ σ*(C-S) ^a^	1.38	1.26	1.09	1.00	1.05	1.05
π(C4-C5)→σ*(C-S)	0.92	1.63	0.33	0.29	0.54	-
π(C1-N)→σ*(C-S)	0.71	0.38	1.51	1.42	0.96	0.75

^a^ Hyperconjugation energy are in kJ/mol.

**Table 10:** Shifts of the ν(C=S) and ν(C=O) vibrations (cm^−1^) in the **A**, **B**, and **C** complexes of pyridines with OCS.

Systems	A Complex		B Complex		C Complex	
	Δν(C=S)	Δν(C=O)	Δν(C=S)	Δν(C=O)	Δν(C=S)	Δν(C=O)
NH_2_-pyr.OCS	−15.9	−20.1	−5.0	3.3	−0.8	−10.0
CH_3_-pyr.OCS	−13.4	−18.6	−3.6	3.2	−2.9	−8.2
Pyr.OCS	−13.6	−17.8	−3.7	3.3	−1.6	−8.1
F-pyr.OCS	−12.6	−16.4	−3.5	3.7	−0.7	−5.5
CN-pyr.OCS	−12.6	−13.9	−3.4	3.5	−0.6	−3.3
NO_2_-pyr.OCS	−11.8	−13.8	−3.5	3.8	−0.6	−2.0

## Supplementary Materials

The following are available online, Figure S1 showing the optimized geometry of the D complex between pyridine and OCS, several correlations presented in S.I. 1, S.I.3, S.I.5, Tables S.I.2, S.I.4, S.I.6 listing SAPT results of the A, B and C complexes and Table S.I.7 presents the Gibb’s free energy values of A, B and C complexes.
