# Supplementary material for: Nature of the Interaction of Pyridines with OCS. A Theoretical Investigation
Source: Molecules. 2020 Jan 19;25(2):416. doi: 10.3390/molecules25020416 (PMC7024555; doi:10.3390/molecules25020416)
Supplement: Supplementary file 1 [file molecules-25-00416-s001.pdf]

Supplementary Information (Pyridine.OCS Complex)

S.I. 1. For the **A** complexes, the following correlations between binding energies and proton affinities (PA) (kJ/mol) or ionization potential (IP) (eV) can be calculated:

-ΔE = 0.023 PA + 10.35 (r^2 = 0.997)

-ΔE = - 1.89 IP -28.40 (r^2 = 0.979)

The slopes of these correlations are somewhat larger than those reported for pyridines.CS_2 systems, respectively 0.019 and -1.57 [46].

Table S.I.2: Results of SAPT analysis for the **A**-complexes between *para*-substituted pyridine and OCS calculated at SAPT2+/aug-cc-pVTZ Level.

Complex	ΔE_{elst}	ΔE_{exch}	ΔE_{ind}	ΔE_{disp}	$\Delta E_{\text{int}}^{SAPT2+}$
NH_2-pyr.OCS	-21.11	29.23	-7.04	-17.82	-16.74
CH_3-pyr.OCS	-20.07	28.06	-6.48	-17.47	-15.96
pyr.OCS	-19.50	27.34	-6.12	-17.12	-15.40
F-pyr.OCS	-18.42	26.32	-5.62	-16.84	-14.56
CN-pyr.OCS	-16.76	24.73	-4.84	-16.56	-13.43
NO_2-pyr.OCS	-16.58	24.39	-4.65	-16.44	-13.28

S.I. 3. For the **B** complexes, the following correlation between binding energies and PA (kJ/mol) and IP (ev) is valuable

-ΔE = 0.009 PA + 2.91 (r^2 = 0.978)

-ΔE = -0.75 IP + 18.17 (r^2 = 0.977)

Table S.I.4: Results of SAPT analysis for the **B-**complexes between *para*-substituted pyridine and OCS calculated at SAPT2+/aug-cc-pVTZ Level.

Complex	ΔE_{elst}	ΔE_{exch}	ΔE_{ind}	ΔE_{disp}	$\Delta E_{\text{int}}^{SAPT2+}$
NH_2-pyr.OCS	-25.22	39.05	-7.35	-24.21	-17.73
CH_3-pyr.OCS	-24.35	37.32	-6.72	-23.64	-17.38
pyr.OCS	-23.68	36.38	-6.37	-23.26	-16.94
F-pyr.OCS	-22.66	35.05	-5.88	-22.82	-16.32
CN-pyr.OCS	-21.76	33.51	-5.15	-22.46	-15.86

NO_2-pyr.OCS	-21.51	32.93	-4.94	-22.29	-15.81

S.I.5. For the **C** complexes, the following correlations between the binding energies and PA or IP are valuable:

-ΔE = 0.0243 PA + 0.34 (r^2 = 0..783)

-ΔE = 2.006 IP + 30.49 (r^2 = 0.786)

Table S.I.6: Results of SAPT analysis for the **C**-complexes between *para*-substituted pyridine and OCS calculated at SAPT2+/aug-cc-pVTZ Level.

Complex	ΔE_{elst}	ΔE_{exch}	ΔE_{ind}	ΔE_{disp}	$\Delta E_{\mathrm{int}}^{SAPT2+}$
NH_2-pyr.OCS	-13.81	32.55	-5.56	-26.66	-13.48
CH_3-pyr.OCS	-14.06	33.63	-5.70	-27.18	-13.31
pyr.OCS	-11.52	28.53	-4.75	-24.02	-11.76
F-pyr.OCS	-10.75	27.99	-4.29	-23.94	-10.99
CN-pyr.OCS	-9.72	28.49	-4.04	-25.09	-10.36
NO_2-pyr.OCS	-9.81	28.77	-4.06	-25.33	-10.42

Table S.I.7: Gibbs Energy values (in kJ/mol) for **A, B** and **C**-complexes of pyridines.OCS systems calculated at the MP2=full/aug-cc-pvTZ//aug-cc-pVDZ Level.

**System**	**A-complex**	**B-complex**	**C-complex**
**NH_2-pyr.OCS**	**10.36**	**10.78**	**5.16**
**CH_3-pyr.OCS**	**11.19**	**11.25**	**4.90**
**Pyr.OCS**	**11.69**	**11.43**	**8.55**
**F-pyr.OCS**	**12.04**	**11.38**	**8.88**
**CN-pyr.OCS**	**12.88**	**11.60**	**5.68**
**NO_2-pyr.OCS**	**12.75**	**11.35**	**4.43**

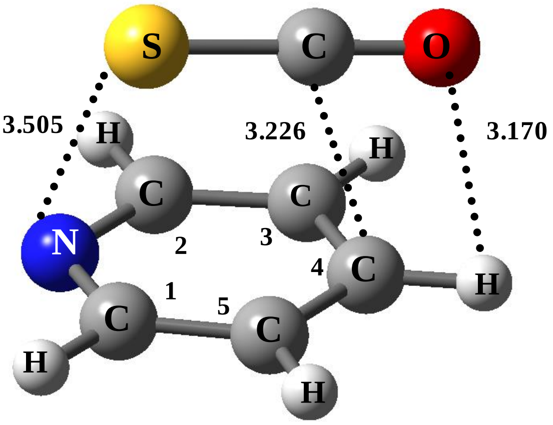

Figure S1: Optimized geometries of the **D** complex between *para*-substituted pyridine with OCS.
